# Anti-proliferation Effect of Polypeptide Extracted from Scorpion Venom on Human Prostate Cancer Cells in vitro

**DOI:** 10.4021/jocmr2009.01.1220

**Published:** 2009-03-24

**Authors:** Yue Ying Zhang, Li Cun Wu, Zhao Peng Wang, Zhao Xia Wang, Qing Jia, Guo Sheng Jiang, Wei Dong Zhang

**Affiliations:** a Key laboratory for Modern Medicine and Technology of Shandong Province, Institute of Basic Medicine, Shandong Academy of Medical Science, Jinan, China;; b Baylor Institute for Immunology Research (BIIR), Baylor Research Institute (BRI), Dallas, Texas, USA.

## Abstract

**Background:**

Prostate cancer is a major cause of cancer-related death in men. Therefore there has been considerable interest to explore neoadjuvant therapy. Polypeptide extracted from scorpion venom (PESV), originally obtained from the East-Asian scorpion Buthus martensi Karsch (BmK), is being studied for both prevention and treatment of various human malignancies including prostate cancer.

**Methods:**

The present study was to investigate the effect of PESV on cell proliferation, cell cycle, and apoptosis in human androgen-independent prostate cancer cells DU-145 in vitro.

**Results:**

PESV treatment on these cells resulted in a significantly dose-dependent growth inhibition with a G1 phase arrest at 40μg/mL after 48h treatment. PESV treatment strongly induced expression of p27 (Kip1), but resulted in a decrease in cyclin E, one of cyclins involved in G1 progression. In other studies, PESV treatment also induced high apoptosis index (AI), confirmed by TdTmediated dUTP-biotin nick-end labeling (TUNEL) assay. Further, the apoptosis induction by PESV (40μg/mL) in DU145 cells was associated with an increase of pro-apoptotic protein Bax.

**Conclusions:**

These results suggest that PESV modulates the expression of cell cycle-related and apoptosis-related proteins and induces growth inhibition and apoptosis of DU145 cells, providing a strong rationale for future studies to evaluate prevention or/and intervention strategies for PESV in pre-clinical prostate cancer models.

**Keywords:**

Prostate cancer, PESV, cell proliferation, cell cycle, apoptosis

## Introduction

Prostate cancer (PCa) is the most invasive and frequently diagnosed malignancy in males in the USA and, at present, is the second leading cause of cancer deaths in North American men [1]. The induction of human PCa has been viewed as a multistage process, involving progression from small, latent carcinomas of low histological grade to large, metastasis carcinomas of higher grade [2-4]. Since the growth and development of PCa is initially androgen-dependent, therefore androgen deprivation has been extensively explored as a strategy for PCa prevention and treatment [4, 5]. While PCa patients treated with androgen-deprivation therapy often have remission within a few years, tumor regrowth occurs, which is largely due to the progression of initially androgen dependent PCa cells to tumor cells that do not depend on androgen for their proliferation [6, 7]. At this stage of the disease, the uncontrolled growth and metastasis potential of malignant cells are extremely aggressive, and unfortunately, the prognosis is death of the patient [6-8]. Hormone refractory prostate cancer (HRPC) remains a challenge in the management of prostate cancer patients. Hence, it would be important to find promising agents against advanced androgen-independent prostate cancer. Chinese medicine which contains many chemical compounds exhibited anti-proliferative effects on cancer cells [9]. Medicinal herbs with anticancer activity have long been used for the treatment or prevention of various human disorders in folk medicine [10-14].

Buthus martensi Karsch (BmK), a species belonging to the Buthidae family, extensively distributed from northwestern China to Mongolia and Korea, is a Chinese herb used for the treatment of various abdominal masses in folk medicine. Polypeptide extract from scorpion venom (PESV), a certain group of polypeptide of 50-60 amino acids extracted from Crude venom of BmK with bioactivities, has been reported to have anti-tumor activity. Our previous results showed that PESV exhibited potent anti-proliferative and apoptosis-induced activity against HUVEC, inhibition of neovascularization, suppression of tumor growth of S180 sarcoma and H22 hepatocelluar carcinoma in mice [15, 16]. In the present study, we tried to look at the effect of PESV on cell proliferation, apoptosis, as well as cell cycle in prostate cancer cells, and further study the possibility of PESV as a potent agent in prevention and/or intervention of human prostate cancer.

Several recent studies by others and us have demonstrated that the cancer preventive and therapeutic efficacy of scorpion venom in different animal tumor models and cell culture systems including prostate, breast, colon and skin cancers [17-23]. Recent studies have begun to elucidate the underlying genetic determinants of the morphologic and biologic characteristics of prostate cancer [24-26]. The molecular and genetic alterations that precede morphological changes and are responsible for tumorigenesis and progression of prostate cancer also include alterations in cell cycle regulators and apoptosis-related proteins causing uncontrolled cancer growth. In general, the progression of cell cycle in eukaryotes is a complex process involving arresting G0 phase, and cell growth involving G1, S and G2/M phases in a step-wise manner [27]. These cell cycle phases receive different growth controlling signals that are integrated and processed for the sequential activation of different members of the cyclin-dependent kinases (CDKs), which are serine/threonine kinases [27-30]. Different CDKs govern different phases of the cell cycle such as G1 by CDK4/CDK6, late G1 to early S by CDK2, and G2/M by p34Cdc2 (CDK1) kinase [27-30]. The kinase activity of CDKs is governed by their regulatory subunits known as cyclins, which form a complex with their catalytic subunit CDKs and are activated at a specific phase of the cell cycle [31-33]. The other important components that control CDK kinase activity are cyclin-dependent kinase inhibitors (CDKIs) Cip1/p21 and Kip1/p27 [31-33]. CDKI is shown to inhibit the kinase activity of CDK-cyclin complexes and thus modulates retinoblastoma (Rb) phosphorylation events, which are essential for various cell cycle transitions [31, 34, 35].

Apoptosis is regulated by several protein families, including the upstream Bcl-2 family (e.g., the anti-apoptotic Bcl-2 and pro-apoptotic Bax) and the downstream caspase family (e.g., caspase-3). The activation of apoptosis is controlled at multiple checkpoints within the cell. Upstream, the pro-apoptotic Bax and anti-apoptotic Bcl-2 are membrane-bound pore-forming proteins that interact through heterodimerization. Together they regulate the mitochondrial transmembrane passage of cytochrome c, which in turn activates caspase proteins. The Bax/Bcl-2 ratio appears more important than the individual Bax or Bcl-2 level in determining a cell's vulnerability to apoptosis; high Bax/Bcl-2 ratios lead to greater apoptotic activity [36].

## Materials and Methods

### Cell culture and treatment

DU145 cells, derived from a brain metastasis of a prostate cancer patient, were purchased from the Institute of Pharmacy, Chinese Academy of Medical Sciences (Beijing, China) and maintained in medium RPMI 1640 supplemented with 10% fetus bovine serum (FBS) in a humidified atmosphere of 5% CO_2_ at 37^o^C. Cells were harvested when they were approximately 70% - 80% confluent. Cells were treated with serial concentrations of PESV in the following experiments. Untreated cells were used as controls.

### Cytotoxicity test of PESV using MTT colorimetric assay

The effect of PESV on the viability of DU145 cells was determined by 3-(4, 5-dimethylthiazol-2-yl)-2, 5-diphenyl tetrazolium bromide (MTT) assay. The cells were plated at 2x10^5^ cells per well in 200μL RPMI 1640 containing 0, 10, 20, 40, 80, 100, and 200 mg/mL PESV in a 96-well microtiter plate. Each concentration of PESV was repeated in 10 wells. The cells were incubated for 48 hours at 37^o^C in an incubator. Following 24 hours of incubation, MTT reagent (4μL, 5 mg/mL in PBS) was added to each well and incubated for 2 hours. The microtiter plate containing the cells was centrifuged at 1,800 rpm for 5 minutes at 4^o^C. The MTT solution was removed from the wells by aspiration and the formazan crystals were dissolved in DMSO (150μL). Absorbance was recorded on a microplate reader at 540 nm wavelength.

### Detection of apoptosis by TUNEL assay

Coverslips with adherent cells treated with PESV (40mg/mL) for 48 hours were fixed in 4% paraformaldehyde for 15 minutes at room temperature. Then rinsed in distilled water and incubated with 0.2% Triton X-100 in phosphate buffered saline solution (PBS)-Tween 20 for 30 minutes. DNA fragments were labeled with TUNEL-Enzyme (Boehringer Mannheim). The kit was used according to the manufacturer's instructions, with the addition of incubation in TdT Reaction Buffer for 10 minutes before TUNEL reaction. The coverslips were then incubated in TdT Reaction Mixture for 2 hours at 40-45^o^C in humidified chamber, rinsed in stop wash buffer for 10 minutes and PBS-Tween 20 for 3 x 2 min. The reaction was detected by incubating coverslips with Streptavidin-HRP in PBS for 20 minutes at room temperature. Then rinse in PBS-Tween 20 for 3 x 2 min, and incubate sections with DAB solution for 5-10 minutes. Cells were counterstained with hematoxylin.

### Assessment of the apoptotic index

A Positive signal was defined as the presence of a dark brown staining on nuclei of the neoplastic cells or on apoptotic bodies as morphologically defined. Cells were defined as apoptotic if the whole nuclear area of cells labeled positively. Apoptotic bodies were defined as small, positively labeled, globular bodies in the cytoplasm of the tumor cells that could be found either singly or in groups. The apoptotic index was determined by the percentage of apoptotic cells divided by the number of tumor cells in 400x magnification. A total of at least 1000 neoplastic nuclei were counted based on 10 randomly chosen fields at 400x magnification [37]. Apoptotic cells were identified by TUNEL in conjunction with characteristic morphological changes, such as cell shrinkage, membrane blebbing, and chromatin condensation.

### DNA cell cycle analysis

The DU145 cells treated with PESV (40 mg/mL) in complete medium for 48 hours. The cells were trypsinized thereafter, washed twice with cold PBS, and centrifuged. The cell pellet was resuspended in 50 μL cold PBS to which cold methanol (450 μL) was added and the cells were incubated for 1 hour at 4^o^C. The cells were centrifuged at 1,100 rpm for 5 minutes, washed twice with cold PBS, suspended in 500 μL PBS, and incubated with 5 μL RNase (20 μg/mL final concentration) for 30 minutes. The cells were chilled over ice for 10 minutes and incubated with propidium iodide (50 μg/mL final concentration) for an additional 30 min in the dark. Cells were then analysed for DNA content using a Becton-Dickinson flow cytometor to determine cell cycle phase.

### Immunohistochemical stainings

Coverslips with adherent cells treated with PESV (40mg/mL) for 48 hours were fixed in 4% paraformaldehyde for 15 minutes at room temperature. DU145 cells were subjected to immunohistochemical staining, with standard streptavidinbiotin- peroxidase techniques, with diaminobenzidine as the chromogen. In brief, DU145 cells were quenched for 10 minutes with 3% hydrogen peroxide, preincubated with blocking serum at 1:20 in 2% bovine serum albumin (BSA)/PBS for 15 minutes at room temperature. After incubation with the primary antibodies, slides were rinsed with PBS, and the secondary antibody was applied at 1:500 in PBS for 30 minutes at room temperature. After rinses with PBS for 30 seconds, slides were incubated with streptavidin/peroxidase at 1:500 in PBS for 30 minutes at room temperature, then rinsed with PBS and incubated for 15 minutes in 0.06% diaminobenzidine and counter-stained with hematoxylin. The following antibodies were used for immunohistochemical staining: Bcl-2 (clone: 124,1:40 dilution, incubation for 2 hours; Dako Corp, Carpinteria, CA), Bax (clone: H62, 1:100 dilution, incubation overnight; Santa Cruz Biotechnology, Inc, Santa Cruz, CA), P27/kip1(clone:M197,1:100 dilution, incubation overnight; Santa Cruz Biotechnology, Inc, Santa Cruz, CA), Cyclin E (clone:M-20,1:50 dilution, incubation overnight; Santa Cruz Biotechnology, Inc, Santa Cruz, CA). Replacing the primary antibody by PBS/5%BSA included negative controls.

### Assessment of protein immunoreactivity by Leica QWin V3 software

Immunoreactivity was quantified within 5 random fields at 100x magnification with a Leica DM4000 B system and Leica QWin V3 software was applied to evaluate grey scale intensity variations. In computing, a grayscale or greyscale digital image is an image in which the value of each pixel is a single sample. Displayed images of this sort are typically composed of shades of gray, varying from black at the weakest intensity to white at the strongest. The grey scale intensity and protein expression have inverse relation.

### Statistics assay

All data are expressed as the mean ± SE. A t test for unpaired or paired data, as appropriate, was used to compare continuous variables between groups. P < 0.05 was considered to indicate statistical significance. All calculations were performed using the SPSS 10.0 statistical package.

## Results

### Inhibition of Cell Proliferation of DU145 cells

**Figure 1:**
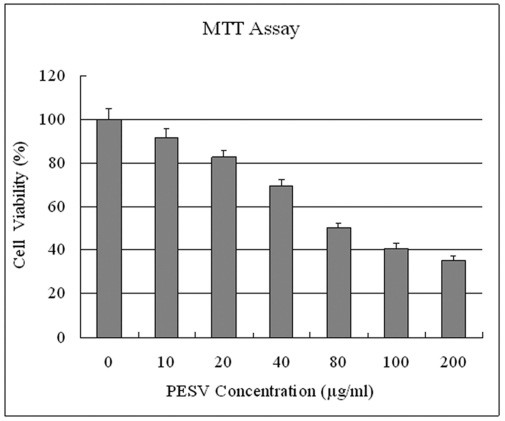
Effect of PESV on the growth of prostate cancer cells DU145. Cells were treated with PESV (10, 20, 40, 80, 100, and 200mg/L), and the percentage inhibition of cell growth was determined by MTT assay in a 96-well microtiter plate as detailed in Materials and Methods. Columns, mean of three separate experiments wherein each treatment was repeated in 10 wells; bars, SE.

We evaluated whether PESV treatment imparts antiproliferative effects in human prostate cancer cells. Employing the MTT assay, we observed that PESV (10- 200 mg/L) treatment on DU145 cells resulted in dose-dependent decrease in the cell growth (Fig. 1).

### Induction of cell apoptosis of DU145 cells and apoptosis index

**Figure 2:**
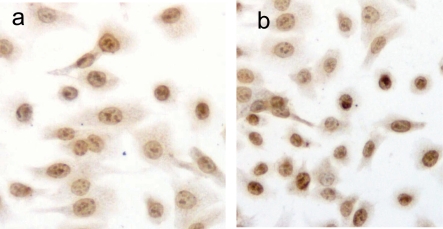
Detection of PESV-induced apoptosis on DU145 cells by DNA fragment TUNEL staining. Apoptotic nuclei and fragmented DNA were stained dark brown in treated cells (2b) but not in untreated controls (2a) in DU145 cells. Original magnification x400.

DU145 cells after exposure to PESV were stained by TUNEL. As shown in Fig. 2, apoptotic nuclei and fragmented DNA were stained dark brown in treated cells (Fig. 2b) but not in the untreated controls (Fig. 2a). The apoptotic index is significantly higher in the PESV-treated cells than in the control (Table 1).

**Table 1:** Effect of PESV on apoptosis in prostate cancer cells DU145 (TUNEL Assay)

Treatment	Apoptosis index (%)	P value
Control	3.6 ± 0.02	
PESV (40 mg/L)	8.3 ± 0.04	0.001

### Apoptosis-related protein expression

**Figure 3:**
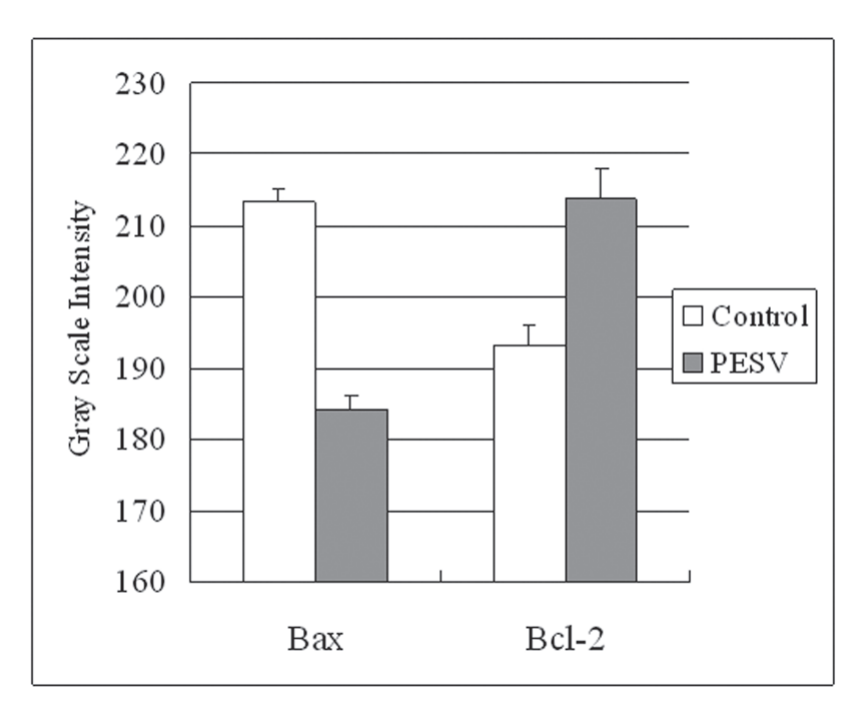
Grey Scale Intensity variants evaluated by Leica QWin V3 software of Bax and Bcl-2 immunoreactivity in DU145 cells treated with PESV and control group. Higher grey scale intensity standing for weaker protein expression, and lower, sronger protein expression. Columns, mean of three separate experiments conducted in triplicate with DU145 cells; bars, SE.

Grey Scale Intensity variants of Bax and Bcl-2 immunoreactivity were evaluated by Leica QWin V3 software in DU145 cells treated with PESV and control group. Fig. 3 indicated the inverse relation between the grey scale intensity and protein expression. Higher grey scale intensity stands for weaker protein expression, and lower intensity stands for stronger protein expression. Treatment with PESV resulted in overexpression of Bax in DU145 cells, whereas Bcl-2 expression reduced. In each comparison, there was a significant difference (P < 0.05).

### Cell cycle analysis and cell cycle-related protein expression

**Figure 4:**
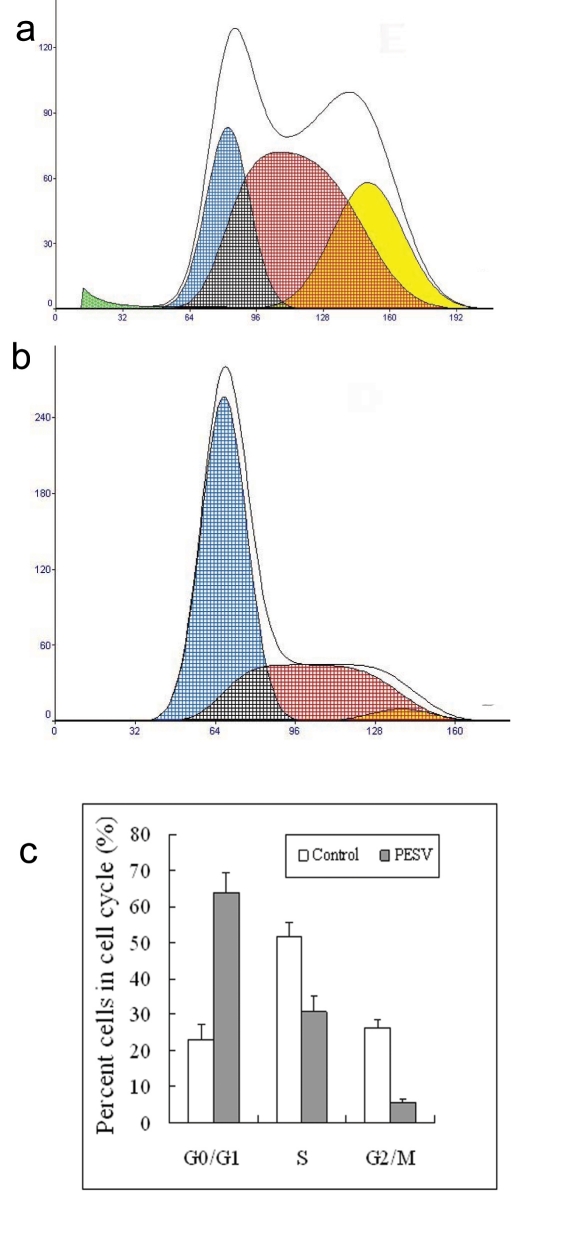
Representative DNA histograms and percentage of cells in different cell cycle phases after incubated with 40mg/L of PESV (B) for 48 h in human DU145 cells by flow cytometry. The growing cells (60% confluent) were treated with PESV (40mg/L) for 48 hours, and the DNA cell cycle analysis was done as described in Materials and Methods. Columns, mean of three separate experiments conducted in triplicate with DU145 cells; bars, SE.

**Figure 5:**
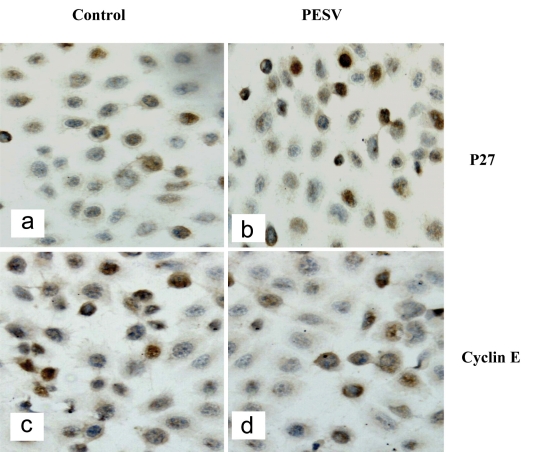
Representative image of cell cycle-related protein expression. P27kip1 (5a, 5b) and Cyclin E (5c, 5d). p27 expressed in the nuclei in DU145 cells treated with PESV (5b) is stonger than the control (5a); But Cyclin E expressed in the nuclei in DU145 cells treated with PESV (5d) is weaker than the control (5c).

**Figure 6:**
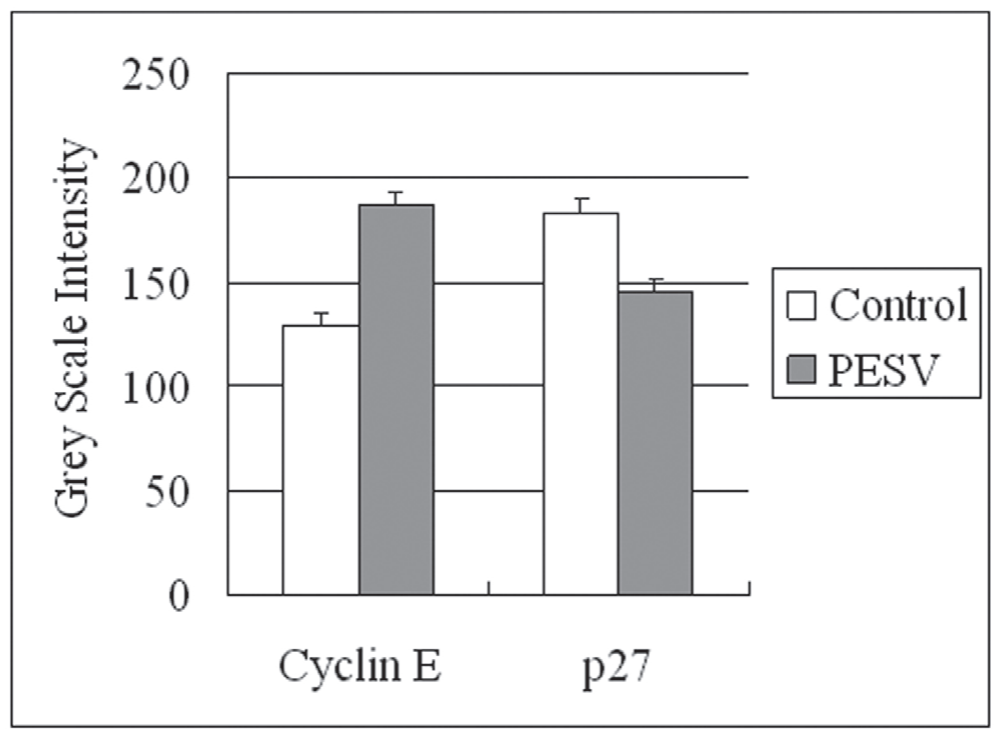
Grey Scale Intensity variants evaluated by Leica QWin V3 software of Cyclin E and p27 immunoreactivity in DU145 cells treated with PESV and control group. Higher grey scale intensity standing for weaker protein expression, and lower, sronger protein expression.

We determined the cell cycle phenotype of DU145 cell line by flow cytometry. As shown in Figure 4, DU145 cells exhibited PESV-associated G1/S arrest occurring at 48 h after exposure to PESV (40 mg/mL). Cells showed a lack of proliferation at 48 h in comparison with untreated controls (not shown), indicating that the PESV (40 mg/mL) caused proliferation arrest. We further detected the expression of cyclerelated protein cyclin E and p27 by immunohistochemical staining. Immunoreactivity was quantified within 5 random fields at 100x magnification. As shown in Fig. 5 and Fig. 6, cyclin E and p27(Kip1) immunostaining pattern was nuclear. DU145 cells exhibited lower expression of cyclin E protein and higher expression of p27(Kip1) protein after exposure to PESV (40 mg/mL) compared with untreated control.

## Discussion

The present study investigates the biological effects of PESV, the major biologically active component in crude scorpin venom, on human prostate cancer DU145 cells. The data support the hypothesis that PESV could be an effective chemoprevention/intervention agent for prostate cancer. Our results clearly demonstrate that PESV induces cell cycle arrest and growth inhibition in human prostate cancer cells, and it induces cell apoptosis at 40 mg/mL concentration with apoptosis index (AI) higher than the controls.

The molecular studies reported here demonstrate that PESV strongly induces the protein expression of Kip1/p27, and decreases cyclin E expression, with an increased interaction/binding between CDKIs and CDK which possibly causes an inhibition in the kinase activity of CDKs. These mechanistic observations were in accordance with an overall efficacy of PESV in inducing a G1 arrest in the cell cycle followed by the inhibition of cell growth.

Cell division depends on the activation of cyclin, which binds to CDKs to induce cell cycle progression towards S phase and later to initiate mitosis; uncontrolled CDK kinase activity is one of the major causes of cancer progression as their functions are tightly regulated by CDKIs such as the Cip1/p21 and Kip1/p27 proteins in normal cell cycle progression [38]. Following anti-mitogenic signals or DNA damage, CDKIs (Cip1/p21 and Kip1/p27) bind to the cyclin–CDK complexes to inhibit their catalytic activity and thus inhibit cell cycle progression [38]. The Rb protein, which is involved in the regulation of the transcription factor E2F [41], is a critical determinant for the restriction-point transition during G1 phase [38, 39], as it is phosphorylated during G1 phase initially by CDK4 or CDK6 and is subsequently maintained in this form by CDK2 [40, 41].

The expression level of cyclins is also an important determinant in cell cycle progression particularly during G1/S and G2/M transitions [38]. D-type cyclins have been shown to be important for progression through the G1 phase, where cyclin E is expressed in late G1 that plays an important role in the G1 to S transition [42-45]. The increased protein expression of G1 cyclins in cancer cells has also been shown to be a major factor in driving uncontrolled growth because cancer cells either lack (with undetectable expression) CDKIs or they are non-functional [46]. The increased expression of CDKIs with decreased expression of cyclins and CDKs and decreased CDK kinase activity induced by PESV treatment in human prostate cancer cells suggest that PESV might be effective for the treatment or prevention of prostate cancer.

Apoptosis is regulated by several protein families, including the upstream Bcl-2 family (e.g., the antiapoptotic Bcl-2 and proapoptotic Bax) and the downstream caspase family (e.g., caspase-3). The activation of apoptosis is controlled at multiple checkpoints within the cell. Bcl-2 family members are important regulators of apoptosis that include antiapoptotic (Bcl-2, Bcl-XL and Mcl-1), proapoptotic (Bax and Bak) and the BH3-domain-only (Bim, Bid and Bik) proteins [47]. Molecules belonging to the B-cell lymphoma leukaemia-2 (Bcl-2)/Bax system play a crucial role in the regulation of the apoptotic process. In particular, Bcl-2 is an intracellular protein that inhibits apoptosis while Bax counteracts the anti-apoptotic function of Bcl-2 by binding to this molecule [36]. Upstream, the proapoptotic Bax and antiapoptotic Bcl-2 are membrane-bound pore-forming proteins that interact through heterodimerization. Together, they regulate the mitochondrial transmembrane passage of cytochrome C, which in turn activates caspase proteins. The Bax/Bcl-2 ratio appears more important than the individual Bax or Bcl-2 level in determining a cell's vulnerability to apoptosis; high Bax/Bcl-2 ratios lead to greater apoptotic activity [36]. This study has also shown that PESV induces cell apoptosis in DU145 prostate cancer cells. We conformed that the induction of apoptosis was mediated through Bax overexpression with bcl-2 down regulation.

Cancer develops when the balance between cell proliferation and cell death is disturbed, and the aberrant cell proliferation leads to tumor growth. It is well known that apoptosis and its related signaling pathways have a profound effect on the progression of cancer [48-49], suggesting that agents inducing apoptotic death of human cancer cells may play a critical role in cancer prevention/intervention including prostate cancer. In this regard, whereas there are several classes of chemotherapy drugs causing apoptotic death of cancer cells, their non-selective efficacy (toxicity) in other tissues has been a limitation in their efficacy. Our data demonstrate significant apoptotic death induction by PESV only in prostate cancers, but not in normal prostate epithelial cells, suggesting the possibility of both selectivity and specificity in PESV efficacy against prostate cancer cells.

More studies, however, are needed in the future to support this assumption, and to identify its mechanism of action in modulating mitogenic and survival signaling cascades in human prostate cancer. In addition, the significance of the observations made in the present study need to be established in a broader context by conducting further studies in a variety of cell lines and xenograft animal models of human prostate cancer. The encouraging data suggest that PESV might be a promising agent in adjuvant therapy for prostate cancer.
